# 1325. Macrolide versus Non-macrolide in Combination with Steroids for the Treatment of Lobar or Segmental *Mycoplasma Pneumoniae* Pneumonia Unresponsive to Initial Macrolide Monotherapy

**DOI:** 10.1093/ofid/ofac492.1155

**Published:** 2022-12-15

**Authors:** Eunha Bae, Hyun Mi Kang, Ye Ji Kim, Dae Chul Jeong, Jin Han Kang

**Affiliations:** The Catholic University of Korea, Seoul, Seoul-t'ukpyolsi, Republic of Korea; The Catholic University of Korea, Seoul, Seoul-t'ukpyolsi, Republic of Korea; The Catholic University of Korea, Seoul, Seoul-t'ukpyolsi, Republic of Korea; The Catholic University of Korea, Seoul, Seoul-t'ukpyolsi, Republic of Korea; The Catholic University of Korea, Seoul, Seoul-t'ukpyolsi, Republic of Korea

## Abstract

**Background:**

*Mycoplasma pneumoniae* (MP) is one of the most common causes of bacterial pneumonia in children. In the recent decade, macrolide-resistant MP (MRMP) has been increasing in proportion, leading to children unresponsive to initial macrolide therapy. The purpose of this study was to evaluate the outcomes of children with lobar or segmental MP pneumonia unresponsive to initial macrolide therapy, who received non-macrolide (NM), macrolide plus steroids (M+S), and non-macrolide plus steroids (NM+S) according to the 2019 guideline during the 2019-2020 Mycoplasma epidemic in South Korea.

**Methods:**

This was a retrospective cohort study of children below 18 years old, admitted during the 2019-2020 MP outbreak for lobar or segmental MP pneumonia. The inclusion criteria were as follows: 1) sputum or nasopharyngeal swab MP PCR positive, 2) no history of pneumonia within one month of positive MP PCR, 3) lobar or segmental pneumonia on chest x-ray, and 4) initial treatment with macrolide monotherapy. Children that were unresponsive to the initial 3-5-day macrolide monotherapy were divided into 3 groups depending on the next regimen: NM, M+S, and NM+S group. Their outcomes were assessed.

**Results:**

During May 2019 to March 2020 MP epidemic, a total 190 patients that fit all of the inclusion criteria were included as study participants. Of these, 16.8% (n=32/190) were responsive to initial macrolide monotherapy and 83.2% (158/190) were unresponsive. Of the 158 patients unresponsive during the initial 5-day macrolide therapy, 8.2% (n=13) were switched to a NM, 75.9% (n=120) were added steroids (M+S), and 15.8% (25/158) were switched to a non-macrolide plus steroids (NM+S). Treatment success rates were 46%, 81%, and 100% in the NM, M+S, and NM+S groups, respectively (*P*=0.001). Compared to patients in the NM+S group, those in the M+S group were 8.0 (Confidence interval [CI], 1.3-61.7; *P*=0.046) times more likely to have prolonged fever ≥4 days after the switch in treatment regimen compared to patients with NM+S, and 10.7 (CI, 1.5-108.7; *P*=0.046) times more likely in the NM group.

**Figure:**
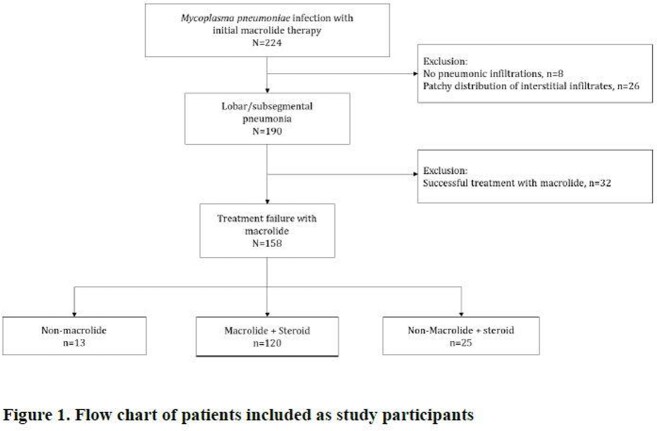

**Figure:**
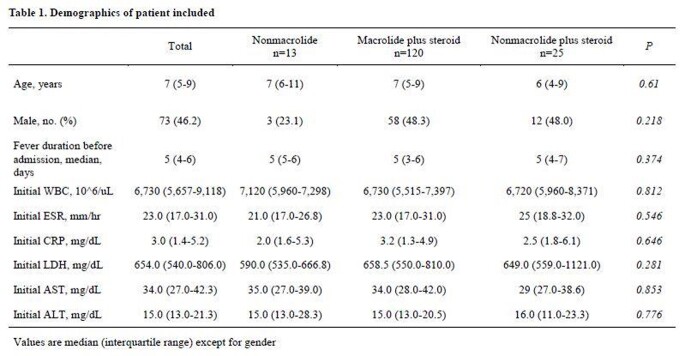

**Figure:**
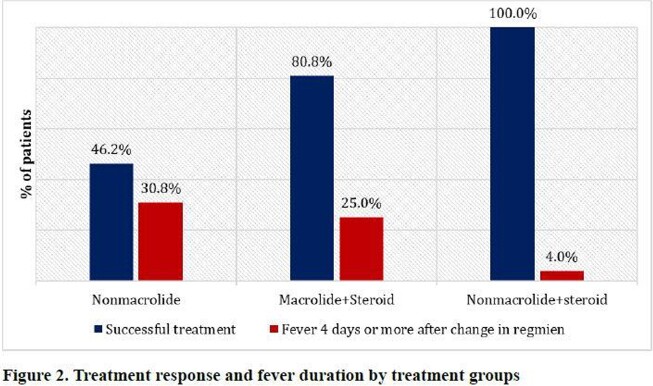

**Conclusion:**

In patients with severe mycoplasma pneumonia with failure of response to initial macrolide therapy, a non-macrolide antibiotic plus steroid combination had the highest treatment success rate and shorter duration of fever.

**Disclosures:**

**All Authors**: No reported disclosures.

